# Fabrication of Electrospun Xylan-g-PMMA/TiO_2_ Nanofibers and Photocatalytic Degradation of Methylene Blue

**DOI:** 10.3390/polym14122489

**Published:** 2022-06-18

**Authors:** Yangyang Xie, Xiao-Feng Sun, Wenbo Li, Junhui He, Ran Sun, Sihai Hu, Yaoguo Wu

**Affiliations:** 1 Shenzhen Research Institute, Northwestern Polytechnical University, Shenzhen 518057, China; yangyang636@mail.nwpu.edu.cn (Y.X.); husihai@nwpu.edu.cn (S.H.); wuygal@nwpu.edu.cn (Y.W.); 2 Research Centre of Advanced Chemical Engineering, School of Chemistry and Chemical Engineering, Northwestern Polytechnical University, Xi’an 710129, China; hjhui@mail.nwpu.edu.cn; 3 Queen Mary University of London Engineering School, Northwestern Polytechnical University, Xi’an 710129, China; liwenbo666@mail.nwpu.edu.cn

**Keywords:** xylan, PMMA, electrospinning, TiO_2_, photocatalytic degradation

## Abstract

Herein, xylan-g-PMMA was synthesized by grafting poly(methyl methacrylate) (PMMA) onto xylan and characterized by FT-IR and HSQC NMR spectroscopies, and the xylan-g-PMMA/TiO_2_ solution was used to electrospun nanofibers at the voltage of 15 Kv, which was the first time employing xylan to electrospun nanofibers. Moreover, the electrospinning operating parameters were optimized by assessing the electrospinning process and the morphology of electrospun fibers, as follows: the mixed solvent of DMF and chloroform in a volume ratio of 5:1, an anhydroxylose unit (AXU)/MMA molar ratio lower than 1:2, the flow speed of 0.00565–0.02260 mL/min, and a receiving distance of 10–15 cm. Diameters of the electrospun fibers increased with increasing DMF content in the used solvent mixture, MMA dosage, and receiving distance. TiO_2_ nanoparticles were successfully dispersed in electrospun xylan-g-PMMA nanofibers and characterized by scanning electron microscopy, energy dispersive X-ray diffraction spectrum, and X-ray photoelectron spectroscopy, and their application for methylene blue (MB) degradation presented above 80% photocatalytic efficiency, showing the good potential in water treatment.

## 1. Introduction

Electrospinning, short for electrostatic spinning, is an old fiber fabrication technique, but now it serves as a convenient and facile method for the preparation of multilevel functional nanometer-scale polymer fibers [1,2], which was observed in 1897 by Rayleigh, studied in detail by Zeleny on electro-spraying in 1914 [3], patented by Formhals in 1934 [4], and finally made tremendous progress in theory and practice due to the efforts of Taylor [5]. Electrospinning, an electrohydrodynamic procedure, is typically considered to be the process of liquid droplets being electrified to generate jets, followed by stretching and elongating to yield fibers [6]. In the electrostatic spinning process, the polymer solution or melt forms a jet in a strong electric field, and the droplet at the tip of the needle will transform from a spherical shape to a conical shape, known as a Taylor cone, which will extend from the tip of the cone to produce a fibrous filament. In this way, polymer filaments of nanometer diameter can be obtained [7,8,9]. Over the past few years, electrostatic spinning has been extensively adopted for the fabrication of various nanofiber materials, out of which the organic polymers in solution or melt form are the most commonly used materials [10]. A wide range of synthetic polymers (polyoxyethylene [11], polylactic acid [12,13], polyacrylonitrile [14], polyamide [15], polycaprolactone [16], polyvinyl pyrrolidone [17], polyvinylidene difluoride [18], polyethersulfone [19]) and natural polymers (collagen [20], chitosan [21], cellulose [22,23], etc.), or a blend of both [11,24], have been applied in electrospinning to prepare nanofibers, as they are soluble or do not degrade at their melting points [1,15]. Nevertheless, compared to synthetic polymers, natural polymers present advantages such as being biodegradable, safe, non-toxic, excellent biocompatibility, economical, and easily available [10,25,26,27,28].

In the aspect of abundant and renewable plant resources, hemicellulose is only less than cellulose in percentage composition and is defined as the group of heteroglycans consisting of xylose, mannose, arabinose, galactose, glucose, and 4-*O*-methyl-D-glucuronic acid residues, which were not widely applied owing to their complex chemical structure and low molecular weight [29,30,31,32]. Xylan hemicellulose is extracted from wheat straw, which is soluble in water and composes 53.58–68.34% of hemicellulose material [33,34,35]. Prevalent studies showed that xylan is composed of a backbone of β-(1→4)-linked hydroxylase units, where the C-2 and C-3 positions are linked with hydroxyl groups or their derivates [33,36]. Due to its newly certified surface-active, bioactive, antimicrobial, biocompatible, and oxygen barrier properties [37,38], considerable interest has been focused on xylan in applications of sewage treatment [39], food packaging [40,41], drug delivery [35,42], and tissue engineering [36,43]. In addition, many organic polymers used in electrospinning have been reported in the past, but there are few reports about xylan electrospinning. Therefore, employing xylan to prepare novel nanofiber materials that combine the properties of xylan itself with the advantages of nanofibers by the electrospinning method is a worthy research direction.

Diameters of electrospun fibers range from 100 nm to 1 μm, which means electrospun fiber mats provide a huge specific surface area with high porosity. Furthermore, the microscopic fine structure of the electrospun fibers is controllable [44,45]. Due to those advantages, electrospun fibers have potential in catalysis [46,47], filtration membrane [48,49], drug delivery [50,51], photocatalysis [52], antibacterial [53], and other applications [54,55]. In recent years, numerous methods have been reported to increase the specific surface area of catalysts for enhancing the efficiency of catalytic reactions [56,57]. A straightforward method of increasing the surface area is to make the catalyst into a nanometer scale. However, this will cause enormous wastage when those nanometer-scale materials are in use due to their difficulty in separating nanometer-scale catalysts from the remaining raw materials and products. A further improvement is loading the catalysts on a porous and hard substrate, which generally is a kind of adsorbing material, such as glasses or ceramics. However, this would decrease the reactive sites, and those inorganic substrates always have some inevitable surficial defects, and the interaction between the catalyst and the substrate is physical and weak. To minimize these negative effects and to expand the available catalytic material resources, nanoscale organic polymer electrospun fibers can be promising candidates for catalysts or their substrates attributed to their fine microscopic nature, huge specific surface area, high porosity, and convenience in preparation [58,59]. Therefore, compounding nanofibers with catalysts is a promising idea to prepare efficient photocatalysts.

In numerous photocatalysts, titanium dioxide (TiO_2_), due to its excellent chemical stability, low cost, non-toxicity, safety, and efficient photocatalytic activity, is used extensively in energy and environmental areas [60,61,62,63,64], such as hydrogen production from decomposing water [65,66,67], pollutant decomposition in water [68,69], and carbon dioxide reduction [70,71,72]. The photocatalytic properties of TiO_2_ depend on the crystalline structure, which modifies its bandgap. In fact, there are three polymorphs: anatase, rutile, and brookite [73,74]. TiO_2_ is one of the most important photocatalytic materials because it is a wide-bandgap semiconductor material (~3.2 eV) suitable only to absorb UV light, and hence photogenerated charge carriers, electrons in the conduction band, and holes in the valance band [63,64]. The electrons are of high reducibility, which can deoxidize the substance absorbed on the surface of TiO_2_, while the holes are of high oxidizability, which can oxidize the absorbed substance by capturing its electrons. When it is applied in photocatalytic degradation, the holes of TiO_2_ oxidize the absorbed water on the surface, producing hydroxide free radicals (•OH), and the electrons of TiO_2_ react with oxygen-producing superoxide free radicals (•O^2−^ and •OOH), which can all violently oxidize adsorbed pollution on the surface of TiO_2_ [75,76,77]. Despite the considerable advances that have been achieved in TiO_2_ in the field of photocatalysis, many problems remain, which presents some attractive challenges. One of the issues that cannot be ignored is that the normal usages of TiO_2_ include powder, coating on the membrane surface, or casting into an alloy block, which all decrease the actual catalytic efficiency of TiO_2_ and are uneconomical for the high cost of separating nanometer-scale catalysts from the remaining raw materials and products. However, loading TiO_2_ on electrospun fibers can solve those problems.

In this study, PMMA was grafted on biopolymer xylan to electrospun nanofibers for the first time. The chemical structural features of the synthesized products were proven by FT-IR and NMR analyses. The factors, including the molar ratio of raw materials, solvent, flow speed, and receiving distance, affected the electrospinning process, and the prepared nanofibers were studied by assessing the operability of the process and the SEM, EDX, and XPS analyses of the electrospun fibers. Nano-TiO_2_-doped xylan-g-PMMA electrospun nanofibers were also obtained, and their photocatalytic activity for MB degradation was measured.

## 2. Materials and Methods

### 2.1. Materials and Chemicals

Xylan used in this study was isolated from wheat straw by the alkaline peroxide method [32], which has a weight-average molecular weight of 18,350 g mol^−1^. The isolated xylan was purified with 75% ethanol-water solution and then dried at 60 °C for 24 h in a vacuum oven. N, N-Dimethylformamide (DMF) and MMA were purchased from J&K Scientific Co., Ltd. in Beijing, China and ammonium persulfate ((NH_4_)_2_S_2_O_4_) and sodium sulfite (Na_2_SO_3_) were purchased from Tianjin Hongyan Chemical Reagent Factory in China. TiO_2_ nanoparticles of 30 nm were supplied by ST-NANO SCIENCE & TECHNOLOGY Co., Ltd., Shanghai, China. All chemical reagents were of analytical grade. 

### 2.2. Synthesis of Xylan-g-PMMA

The xylan-g-PMMA were synthesized from xylan and MMA by using ammonium persulfate ((NH_4_)_2_S_2_O_4_) and sodium sulfite (Na_2_SO_3_) as a catalytic system. Xylan (2000 mg) was first dissolved in deionized water (40 mL) at room temperature, and subsequently, DMF (40 mL) was added. Then, the mixture was placed in a rotary evaporator to remove the deionized water at 50 °C. After adding lithium chloride (400 mg), it was stirred to acquire a homogeneous solution. For the synthesis of xylan-g-PMMA, the xylan solution was kept in a 50 °C water bath, in which (NH_4_)_2_S_2_O_4_ (50 mg) and Na_2_SO_3_ (50 mg) were added, stirring for 5 min. MMA was added to the mixture at different AXU/MMA molar ratios of 1:2, 1:4, 1:6, and 0:10 and stirred for 6 h at 50 °C. At the end of the reaction, the mixture was poured slowly into excess ethanol and filtered. This was followed by drying the product in vacuo for 12 h to obtain a solid.

### 2.3. Viscosity Measurement and Structural Characterizations

Xylan-g-PMMA (500 mg) was mixed with different ratios of xylan: MMA was dissolved in a mixture of DMF (5 mL) and chloroform (1 mL) at room temperature, respectively. Shear viscosity (η) values for the xylan-g-PMMA solutions were obtained from an NDJ-79A rotational viscometer (Changji Geological Instrument Co., Ltd., Shanghai, China) at room temperature.

FT-IR spectra of the xylan and xylan-g-PMMA were recorded by a Nicolet 510 spectrophotometer (Thermo Scientifific, Waltham, MA, USA) in the 4000–400 cm^−1^ region. ^1^H, ^13^C heteronuclear single-quantum coherence (HSQC) NMR spectra were recorded by a Bruker AVANCE spectrophotometer (400 MHz), and 50 mg of dried xylan-g-PMMA with AXU/MMA at a molar ratio of 1:4 was dissolved in 0.5 mL of dimethyl sulfoxide-d_6_ (DMSO-d_6_) at 40 °C and recorded at room temperature. All chemical shifts were relative to the resonance of tetramethylsilane (TMS, δ = 0). Electrospun fibers’ images were obtained by using a VEGA 3 LMH SEM microscope (TESCAN, Brno, Czech Republic) at 10 kV after being transferred to an SEM stud and coated with gold.

### 2.4. Electrospinning

Electrospun fibers were fabricated by a 500 mg xylan-g-PMMA solution using an SS-2534H electrospinning device (Ucalery, Co., Ltd., Beijing, China) at 15 kV under room temperature and 20% humidity. Various xylan-g-PMMA solutions were placed in a 5 mL syringe with a metal needle (0.81 mm × 0.51 mm), respectively. Electrospun fibers were collected on aluminum foil.

### 2.5. The Preparation of Electrospun Xylan-g-PMMA/TiO_2_ Fibers

TiO_2_ nanoparticles were added to the solutions of xylan-g-PMMA with different mass ratios of 0%, 10%, and 30%. To reduce the loss of TiO_2_ loaded on the electrospun fibers in the further application, triethoxyvinylsilane was added into the spun liquid in the mass ratio of 62%, to TiO_2_. The well-dispersed mixtures were electrospun at the optimal condition for 30 min according to Section 2.4. The electrospun fibers were obtained on aluminum foil and characterized by the VEGA 3 LMH SEM microscope and INCA X-ACT energy dispersive spectroscopy (Oxford, UK).

### 2.6. Photocatalytic Degradation Property of Electrospun Xylan-g-PMMA/TiO_2_ Fibers

MB aqueous solutions of 0.1, 0.2, 0.3, 0.4, and 0.5 mg/L were prepared, and their absorbance values were measured by an ultraviolet spectrophotometer (752B, Tianjin Precise Instrument Co., Ltd., Tianjin, China) at 665 nm. The standard curve was obtained by linear-fitting the values, A=0.094c+0.0402, and the linear correlation coefficient was 0.99759.

Then, 20 mg/L of MB aqueous solution was used for photocatalytic degradation. The electrospun xylan-g-PMMA/TiO_2_ fibers collected on aluminum foil were cut into the mats of 6 × 2.5 cm^2^, then put into 30 mL of MB aqueous solution under the irradiation of 125 W ultraviolet light (365–400 nm; Ausbond, China) with a distance of 5 cm between the light and the surface of the solutions. The absorbance values of the MB aqueous solutions were detected at 665 nm every 30 min by using the ultraviolet spectrophotometer. The MB photocatalytic degradation rate (DR) was calculated by the equation of $DR=(c_{0}-c)/c_{0}$, where *c*_0_ stands for the initial concentration of the MB aqueous solution.

## 3. Results

### 3.1. FT-IR Analysis

**Figure 1:**
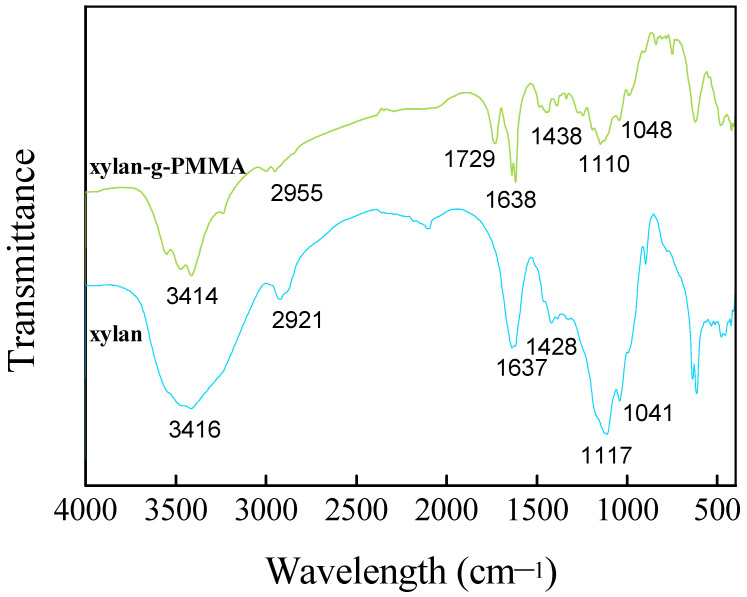
FT-IR spectra of xylan and xylan-g-PMMA.

Due to the vibration or rotation of different functional groups and chemical bonds, they absorb infrared light at different wavelengths. Accordingly, FT-IR can be used to determine which functional groups or chemical bonds are present or changed in a sample, and for qualitative, quantitative, and reaction process studies of substances [78,79]. To further understand the chemical bond and group changes of xylan and xylan-G-PMMA, the samples were analyzed by FT-IR. The FT-IR spectra of xylan and xylan-g-PMMA (the AXU/MMA molar ratio is 1:4) are shown in Figure 1. The infrared spectrum of xylan polymer displays a broad absorption band at 3419 cm^−1^ that is attributed to OH stretching associated with polar groups linking through intra- and inter-molecular hydrogen bonding [80]. The absorptions around 2921 and 1428 cm^−1^ correspond to C-H stretching and bending vibration of CH_2_ in the molecule. The absorption at 1637 cm^−1^ owes to the absorbed water in the sample. The absorption at 1117 cm^−1^ arises from the stretching vibration of C-C in the xylan polymer, and the absorption at 1041 cm^−1^ results from the bending vibration of C-OH [81]. These main characteristic absorptions were also observed in the infrared spectrum of the xylan-g-PMMA. Particularly, the absorption peak at 1729 cm^−1^ was typically observed in the xylan-g-PMMA spectrum, which is associated with C=O stretching of the methoxycarbonyl group [82,83]. All these results indicated the successful graft copolymerization of xylan and MMA.

### 3.2. NMR Analysis

**Figure 2:**
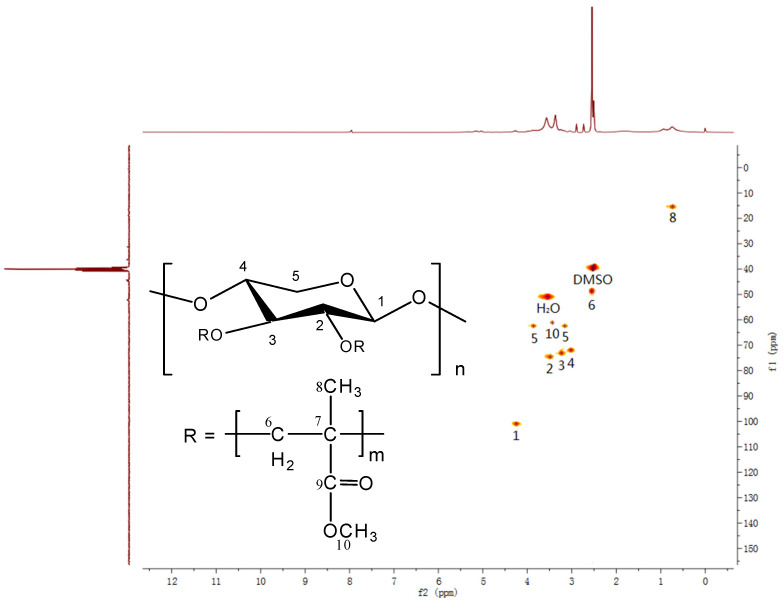
^1^H-^13^C HSQC NMR spectrum of xylan-g-PMMA.

Figure 2 presents the ^1^H-^13^C HSQC NMR spectrum of the xylan-g-PMMA (the AXU/MMA molar ratio is 1:4), and the vertical axis (f1) denotes ^13^C NMR chemical shifts (δC) while the horizontal axis (f2) denotes ^1^H NMR chemical shifts (δ_H_). As marked in Figure 2, the attributions of those signals were: (δ_C_,δ_H_) (100.94, 4.20): (C1, H); (74.91, 3.44): (C2, H); (73.40, 3.19): (C3, H); (71.87, 2.97): (C4, H); (62.58, 3.84/3.19): (C5, H_a_/H_b_); (45.59, 2.50): (C6, H2); (15.34, 0.68): (C8, H3); (61.07, 3.41): (C10, H3) [33,84,85]. The strong signals at (39.52, 2.56) ppm and (51.00, 3.53) ppm were derived from the solvent DMSO and the absorbed water in the sample, respectively. No protons connected with C7 and C9 resulted in no signals shown in the 2D spectrum in Figure 2. The chemical shifts were recorded at 44.32 and 177.70 ppm in the ^13^C NMR spectrum, corresponding to C6 and C9, respectively. These typical signals detected in the HSQC NMR spectrum demonstrated the targeted synthesis of xylan-g-PMMA.

### 3.3. Polymer Solution Viscosity

**Figure 3:**
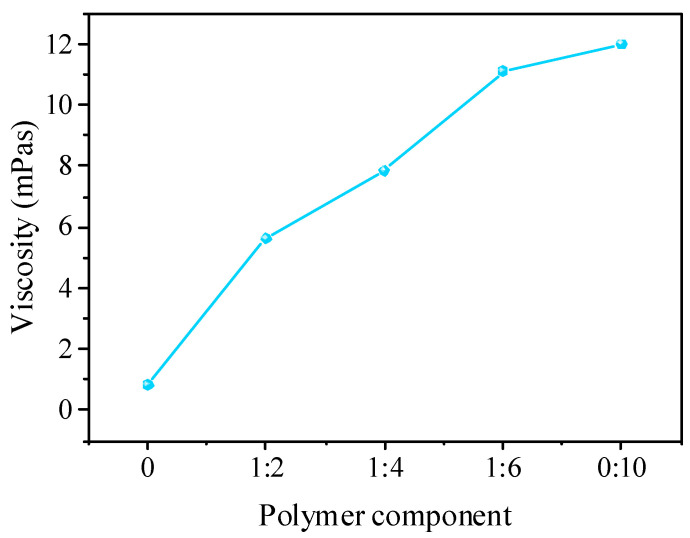
The viscosity of xylan-g-PMMA solutions with different AXU/MMA molar ratios.

Various xylan-g-PMMA solutions with AXU/MMA molar ratios of 1:2, 1:4, 1:6, and 0:10 were prepared in the mixed solvent of DMF and chloroform, with the weight concentration of 7.42%. The viscosity data of the solutions at room temperature are shown in Figure 3. It illustrates that, when increasing MMA is added to the polymerization reaction, the solutions would become stickier. As the viscosity of a polymer (solution) has a strong positive correlation with its molecular weight, it can be concluded that the increase of added MMA content in the reaction contributed to the increase of the molecular weight of the products.

### 3.4. Effect of Solvents on Electrospinning

**Figure 4:**
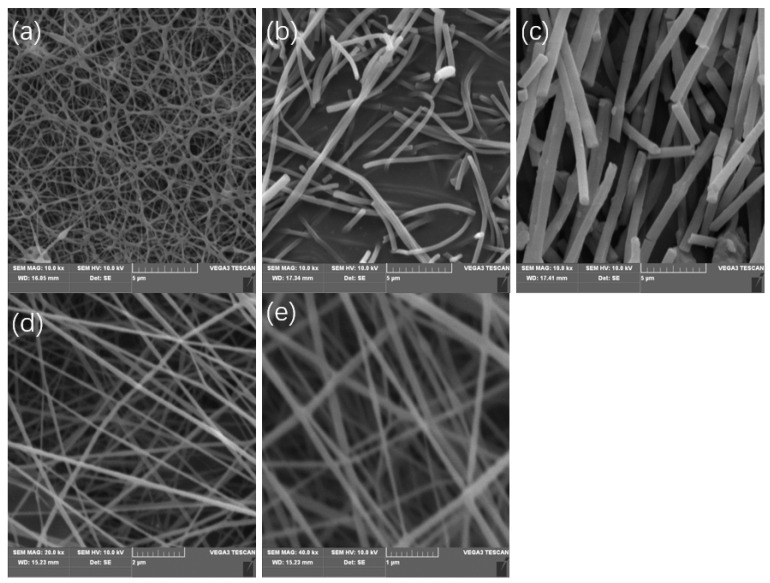
The SEM images of the fibers produced with the solvent of (**a**) pure DMF, (**b**) 5 mL of DMF + 2 mL of acetone, (**c**) 5 mL of DMF + 1 mL of acetone, (**d**) 5 mL of DMF + 1 mL of chloroform + 1 mL of acetone, and (**e**) 5 mL of DMF + 1 mL of chloroform. (**a**–**c**) 10 kx, (**d**,**e**) 20 kx.

The solvent is essential to solution electrospinning. As stated in the Introduction Section, the solvent should be equipped with appropriate volatility, with the boiling point in the range of 70 to 150 °C, in a normal experimental situation without any other remedies. Acetone had been tried in this study, which led to the interruption of the process since the needle of the syringe was jammed by the solidified copolymer. As a result of its good volatility, acetone in the flow evaporated too fast to keep enough fluidity in the flow before it was sprayed. DMF had been attempted as well because the xylan-g-PMMA could dissolve in it well. Instead of individual fibers, there were only some copolymer nets obtained, as shown in Figure 4a. This may be due to two reasons: the DMF solvent evaporated too slow to obtain the fibers with enough solidity and they became aggregated, and the large surface area of the fiber mats resulted in the excellent adsorption capacity of the solvent vaporized around the surface of the fiber mats, which made it possible for the redissolution of the fibers before the solvent was exhausted from the relatively isolated experimental equipment.

To improve the volatility of DMF and prepare individual fibers, the mixed solvents were obtained by adding acetone to DMF in the volume ratio of 2:5, and the SEM image of the electrospun fibers is shown in Figure 4b. As can be seen, the fibers were broken into pieces. This may be the consequence of too much acetone added, as the fibers were solidified so much that they were easily broken. When decreasing the acetone added in DMF to a volume ratio of 1:5, the surface of the fibers became worse, being fragile and uneven, as shown in Figure 4c. This suggests that acetone may contribute to the dissolution of the product in the mixed solvent. Another solvent with a low boiling point, though higher than acetone, chloroform, was also attempted. When chloroform was added into the DMF in the volume ratio of 1:5, those problems were eliminated, as shown in Figure 4d,e.

In the comparison of the diameters of the electrospun fibers in Table 1, it is found that the diameters decreased with increasing the added solvent ratio with a low boiling point: acetone or chloroform. This can be explained because adding more solvents into the solution reduced the concentration of the copolymer in solvents, which contributed to the decrease of the surface tension of the solutions. The less surface tension affecting the flow, the more intensively the fibers would be stretched. As an additive for DMF solution electrospinning, chloroform is more efficient than acetone to obtain fibers with enough tenacity and small diameters.

### 3.5. Effect of AXU/MMA Molar Ratio on Electrospinning

**Figure 5:**
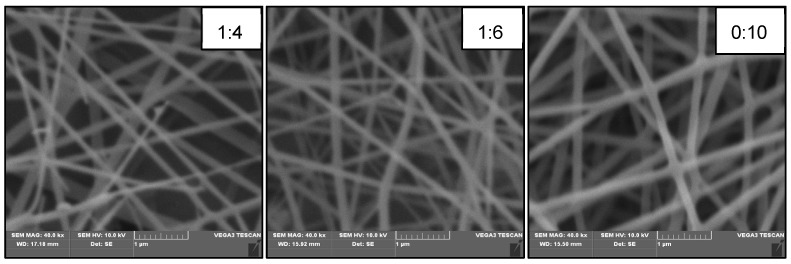
The SEM images (40 kx) of the electrospun fibers were produced with AXU/MMA molar ratios of 1:4, 1:6, and 0:10.

The molar ratio of AXU/MMA primarily has an impact on the molecular weight of the xylan-g-PMMA, which would remarkably affect the electrospinning process. The electrospinning process could not be conducted using the pure xylan or the xylan-g-PMMA with the minimal AXU/MMA molar ratio (1:2), and there were no fibers besides spray-deposited on the aluminum foil. When increasing the MMA ratio added in the polymerization reaction to the molar ratios of 1:4 and 1:6, plentiful fibers were collected on the foil, and the diameters of the fibers increased as well (Table 2 and Figure 5). In an extreme situation with a molar ratio of 0:10, the diameter of the fibers became the largest. These results correspond to the viscosity data of the polymers in Section 3.3. The more MMA added to the reaction, the larger the molecular weight of the products, and the internal resistance of the polymer solutions would become stronger against the electric field force, thus leading to the increase of the diameters of the fibers.

### 3.6. Effect of Flow Speed on Electrospinning

In the continuous electrospinning process, flow speed is an important operating parameter, which should be matched with the flow spraying speed in the high-voltage field, and the latter is correlated to the electrospinning voltage. In this study, the flow speed was set at different values, while the applied voltage was 15 kV. When the speed was lower than 0.00565 mL/min, the electrospinning spray became unstable as no sufficient flow was provided. When the speed was higher than 0.02260 mL/min, there were some fibers dropped from the needle of the syringe as there were too many liquids spraying to the receiver. However, in the tolerable range, no remarkable variations were observed on the fibers as the speed accelerated (Table 3).

### 3.7. Effect of Receiving Distance on Electrospinning

The receiving distance could influence the electrostatic attraction of the flow, which would negatively affect the diameters of the fibers. It was found that when the receiving distance was less than 10 cm, the flow sprayed to the foil directly and no fibers were obtained as the electrostatic attraction was too strong. When the distance was beyond 15 cm, the weak attraction made it possible for the fibers to move with the flow of the air, and the fibers deposited unevenly on the receiving foil on a large scale, which was harmful to the collection of the fibers. After the comparison of the three different conditions (in Table 4), it could be concluded that the diameters of the fibers increased slightly with the increase of the acceptable receiving distance, which is consistent with the former study [86].

### 3.8. Photocatalytic Degradation of MB by Electrospun Xylan-g-PMMA/TiO_2_ Fibers

**Figure 6:**
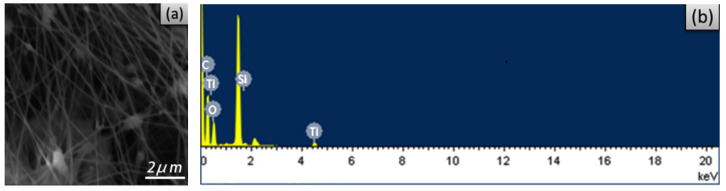
SEM image (**a**) and EDX spectrum (**b**) of electrospun xylan-g-PMMA/TiO_2_ fibers.

According to the above discussions, electrospun xylan-g-PMMA/TiO_2_ fibers were prepared at the following electrospinning parameters: the DMF and chloroform mixed solvent in a volume ratio of 5:1, receiving distance of 10 cm, and flow speed of 0.00565 mL/min. Figure 6a presents the SEM image of the electrospun xylan-g-PMMA/TiO_2_ fibers, and it shows that the electrospun xylan-g-PMMA/TiO_2_ nanofibers were well-prepared, and the presence of some nodes could be considered to be the aggregated TiO_2_ linked with triethoxyvinylsilane, which was proven by the X-ray EDS spectrum (Figure 6b). There is the presence of a Si signal in the EDS spectrum, which was due to triethoxyvinylsilane. To reduce the loss of TiO_2_ loaded on the electrospun fibers, triethoxyvinylsilane was added into the spun liquid.

**Figure 7:**
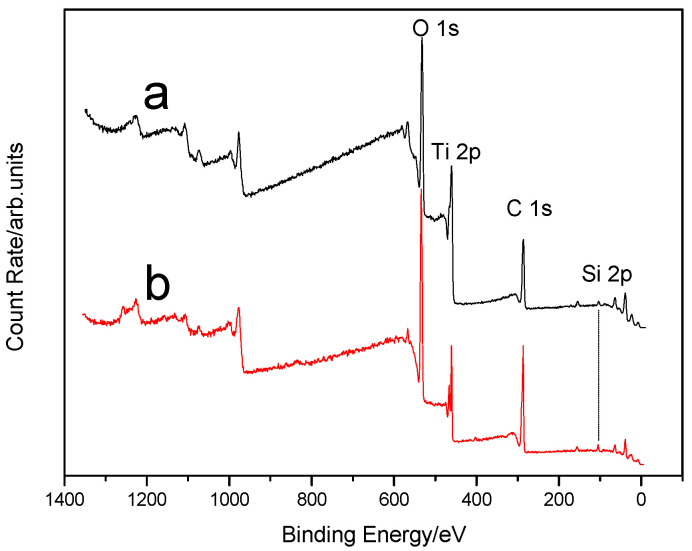
XPS spectra of electrospun xylan/TiO_2_ fibers (a, 10%; b, 30%).

**Figure 8:**
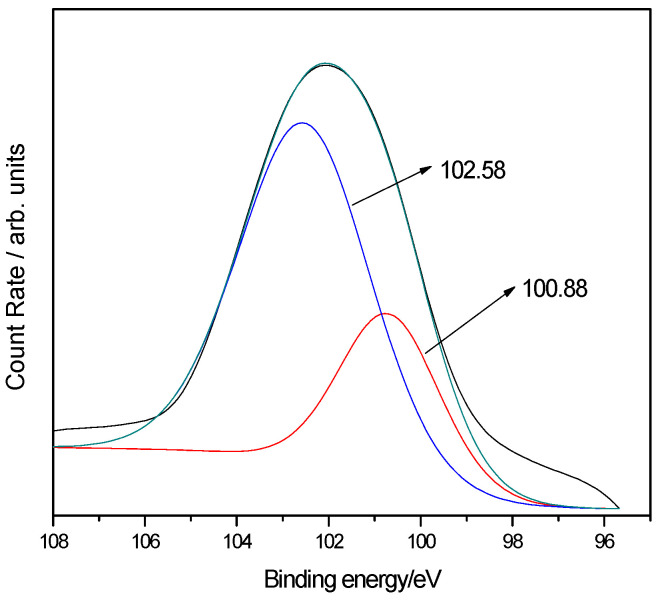
XPS spectrum of Si2p.

**Figure 9:**
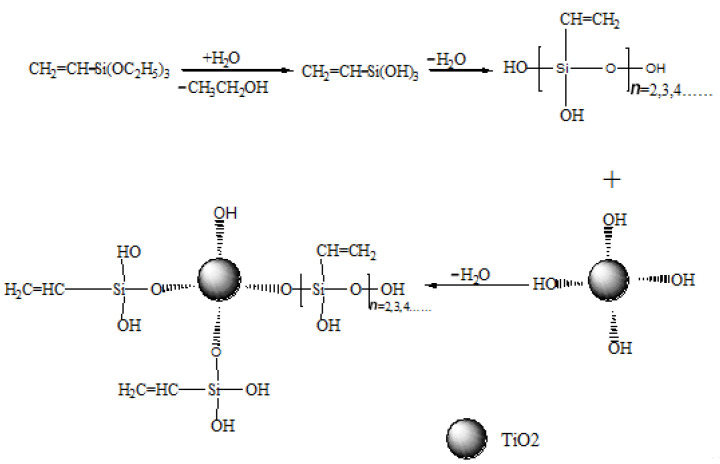
The reaction diagram of TiO_2_ nanoparticles with silane coupling agent.

XPS analysis of electrospun xylan-g-PMMA/TiO_2_ fibers was also performed (Figure 7), and it identified the peaks of Ti, Si, O, and C. The XPS spectrum of Si2p of electrospun xylan-g-PMMA/TiO_2_ fibers showed that Si mainly existed in two forms (Figure 8). The two peaks of binding energy, EB, at 102.58 and 100.88 eV corresponded to Si-O and Si-C peaks [87,88], respectively, indicating that triethoxyvinylsilane reacted with O atoms on the surface of TiO_2_ (Figure 9). This is because the surface of nano-TiO_2_ contains a large number of hydroxyl groups, and the -Si(OH)_3_ generated by the hydrolysis of -Si(OC_2_H_5_) in triethoxyvinylsilane can react with hydroxyl groups, making triethoxyvinylsilane chemically bonded to the surface of nano-TiO_2_ to form Ti-O-Si bonds.

**Figure 10:**
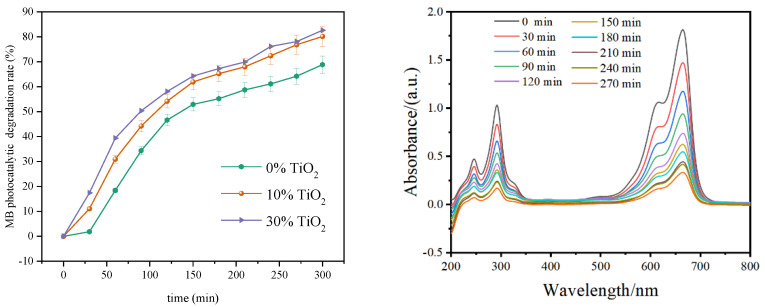
Photocatalytic degradation/removal of MB by the electrospun xylan-g-PMMA/TiO_2_ fibers (a: 0% TiO_2_; b: 10% TiO_2_; c: 30% TiO_2_). The left figure is the UV-vis spectra for the MB solution alone and in the presence of the catalyst during the entire photodegradation process, corresponding to the trends.

The photocatalytic degradation property of the xylan-g-PMMA/TiO_2_ fibers was evaluated, and the photocatalytic degradation of MB is illustrated in Figure 10. According to the obtained results, the electrospun xylan-g-PMMA/TiO_2_ fibers exhibited a better photocatalytic degradation property for MB dye. When the dosage of loaded TiO_2_ increased to 30%, the fiber mat showed the best photocatalytic degradation rate (above 80%), and the superiority was obvious during the first 150 min. It can be concluded that increasing the dosage of loaded TiO_2_ contributed to the increase of MB degradation rates, and MB molecules were degraded faster in the first 150 min (Figure 10), and then the MB photocatalytic degradation rates of the fiber mats loaded with TiO_2_ became slow. Additionally, it was observed that the fiber mats with 10% TiO_2_ worked better since its quantity is 1/3 of the other, and the increase in the amount of TiO_2_ did not correspond to a clear improvement in the photoactivity of the system, which may be due to the superficial segregation phenomena that arose from more TiO_2_ nanoparticles. In the SEM image (Figure 6), it was observed that TiO_2_ nanoparticles aggregated in large nodes and were not homogeneously distributed on the surface of the nanofibers; therefore, the active surface decreased. To summarize, this kind of electrospun xylan-g-PMMA fiber with 10% TiO_2_ can be applied as a promising functional material for water treatment.

## 4. Conclusions

Xylan is an important bioactive polysaccharide in nature, but the fabrication of electrospun xylan fibers is very difficult because of its low molecular weight and solubility. To prepare electrospun xylan fibers, xylan-g-PMMAs were synthesized by copolymerization of MMA and xylan with a catalytic system of (NH4)_2_S_2_O_4_ and Na_2_SO_3_, and electrospun fibers were prepared by solution electrospinning of xylan-g-PMMAs. The mixture of DMF and chloroform in the volume ratio of 5:1, the AXU/MMA molar ratio lower than 1:2, the flow speed of 0.00565–0.02260 mL/min, and the receiving distance of 10–15 cm were found as the optimum operating parameters for electrospinning at the voltage of 15 kV by assessing the electrospinning process and the morphology of the fibers, and the diameters of the fibers increased with increasing the MMA content added in the copolymerization, the DMF content in the solvent, and the receiving distance.

TiO_2_ nanoparticles were loaded in the electrospun xylan-g-PMMA nanofibers at the electrospinning parameters: DMF and chloroform mixed solvent in the volume ratio of 5:1, receiving distance of 10 cm, and flow speed of 0.00565 mL/min. The SEM image and X-ray EDS spectrum proved the successful loading of TiO_2_ on the fibers. The xylan-g-PMMA/TiO_2_ fibers showed good potential in photocatalytic degradation of MB dye. Additionally, this work demonstrates the first use of xylan to fabricate the nanofibers by electrospinning, which will provide a new direction to prepare nanomaterials with xylan. Furthermore, it may offer a novel strategy for the utilization of natural polymers to synthesize high efficiency, environmental, and economic photocatalysis for water treatment.

## Figures and Tables

**Table 1:** The effect of solvents on the diameters of the electrospun fibers.

Molar Ratio of Xylan/MMA	Flow Speed/(mL/min)	Receiving Distance/cm	Solvents	Diameters of the Fibers/nm		
				Max.	Min.	Mean
1:4	0.05650	15	5 mL DMF + 1 mL acetone (c)	1184.83	596.61	744.52
			5 mL DMF + 2 mL acetone (b)	435.57	150.35	297.82
1:4	0.01130	10	5 mL DMF + 1 mL chloroform(e)	160.83	66.28	140.37
			5 mL DMF + 1 mL chloroform + 1 acetone (d)	130.82	63.05	99.41
1:6	0.00565	15	5 mL DMF + 1 mL chloroform	253.35	88.88	164.2
			5 mL DMF + 1 mL chloroform + 1 mL acetone	209.27	88.92	137.41

**Table 2:** The effect of AXU/MMA molar ratios on the diameters of the electrospun fibers.

Molar Ratio of Xylan/MMA	Solvent	Receiving Distance/cm	Flow Speed/(mL/min)	Diameters of the Fibers/nm		
				Max.	Min.	Mean
1:4	5 mL DMF + 1 mL chloroform	15	0.00565	179.79	70.72	125.25
1:6				253.35	88.88	164.2
0:10				362.25	162.3	227.95

**Table 3:** The effect of flow speed on diameters of the fibers.

Molar Ratio of Xylan/MMA	Solvent	Receiving Distance/cm	Flow Speed/(mL/min)	Diameters of the Fibers/nm		
				Max.	Min.	Mean
1:4	5 mL DMF + 1 mL chloroform	10	0.00565	162.79	58.90	111.90
			0.01130	140.85	68.28	108.22
			0.02260	160.83	66.28	109.36
1:6	5 mL DMF + 1 mL chloroform	15	0.00565	267.74	137.4	177.99
			0.01130	320.59	100.0	177.84
			0.02260	291.25	106.9	172.07

**Table 4:** The effect of receiving distance on diameters of the electrospun fibers.

Molar Ratio of Xylan/MMA	Solvent	Flow Speed/(mL/min)	Receiving Distance/cm	Diameters of the Fibers/nm		
				Max.	Min.	Mean
1:4	5 mL DMF + 1 mL chloroform	0.01130	10	160.83	66.28	109.36
			15	211.07	100.11	144.90
1:6	5 mL DMF + 1 mL chloroform	0.01130	10	194.77	84.36	122.72
			15	289.62	91.03	143.16
0:10	5 mL DMF + 1 mL chloroform	0.00565	10	391.64	138.68	251.02
			15	451.96	144.81	296.17

## Data Availability

The data presented in this study are available upon request from the corresponding author.
